# Bevacizumab-containing regimens after cetuximab failure in Kras wild-type metastatic colorectal carcinoma

**DOI:** 10.3892/ol.2012.1045

**Published:** 2012-11-26

**Authors:** KA ON LAM, VICTOR HO FUN LEE, RICO KIN YIN LIU, TO WAI LEUNG, DORA LAI WAN KWONG

**Affiliations:** 1 Department of Clinical Oncology, Faculty of Medicine, The University of Hong Kong;; 2 Department of Clinical Oncology, Queen Mary Hospital, Hong Kong, SAR, P.R. China

**Keywords:** bevacizumab, cetuximab, metastatic colorectal carcinoma, Kras wild-type

## Abstract

Bevacizumab and cetuximab both improve treatment efficacy when administered with chemotherapy for metastatic colorectal carcinoma (mCRC). Cetuximab has enhanced efficacy in Kras wild-type tumors. However, inferior outcomes have been demonstrated concerning the concurrent use of bevacizumab and cetuximab with chemotherapy. There is an urgent need to define the optimal sequence of use of these two agents. With regard to the pre-clinical data that increased VEGF expression is associated with acquired resistance to anti-EGFR antibody, we performed a retrospective analysis on the outcomes of patients who received bevacizumab-containing regimens after cetuximab failure in Kras wild-type mCRC. From January 2006 to December 2011, patients who received bevacizumab-containing regimens for mCRC in our institution were reviewed. Patients were eligible for further analysis if the following criteria were met: i) Kras wild-type mCRC; ii) chemotherapy and cetuximab received as immediate prior treatment; iii) chemotherapy and bevacizumab received as the index line of treatment; and iv) imaging conducted for response evaluation. Outcome measures included median progression-free survival (mPFS) and objective response rate (ORR). Targeted adverse events were recorded in accordance with two prospective observational cohort studies; the BRiTE and BEAT studies. Fifty patients who received bevacizumab-containing regimens were reviewed and 18 of them met the criteria for further analysis. After a median follow-up of 12.1 months, the mPFS for the total group of patients was 26.3 weeks (95% CI, 19.5–33.0 weeks) with an ORR of 38.9%. Two patients (11.1%) had hypertension that required additional anti-hypertensive drugs and one patient did not survive due to a bowel perforation. No arterial thromboembolic events (ATEs), post-operative wound-healing complications (POWHCs) or grade III/IV bleeding were observed. In patients with Kras wild-type mCRC, bevacizumab-containing regimens following cetuximab failure have modest activity and manageable toxicity.

## Introduction

Colorectal carcinoma is one of the most common malignancies worldwide. Prior to the era of targeted therapy, chemotherapy was the only option of systemic therapy for metastatic colorectal carcinoma (mCRC). Chemotherapy regimens, comprising 5-fluorouracil (5-FU) plus leucovorin (LV) backbone with either oxaliplatin or irinotecan, have improved both progression-free survival (PFS) and overall survival (OS) (1–4). Capecitabine, an oral antimetabolite that is metabolised preferentially in tumor cells, in combination with oxaliplatin demonstrates a similar efficacy to FOLFOX4 (oxaliplatin in combination with 5-FU and LV) in the treatment of mCRC (5).

The development of targeted therapy has expanded treatment options for patients with mCRC. Bevacizumab, a recombinant humanized monoclonal antibody against vascular endothelial growth factor (VEGF), used in conjunction with chemotherapy has demonstrated efficacy as a first- and second-line treatment (6–8). On the other hand, cetuximab, a chimeric monoclonal antibody against epithelial growth factor receptor (EGFR), is active as a first- and second-line treatment when combined with chemotherapy and as a single agent in third-line therapy (9–11). It has enhanced the effect of treatment in patients with Kras wild-type mCRC (9,12) and testing for the Kras status of the tumor specimen is recommended in various international treatment guidelines.

With regard to chemotherapy, the efficacy is independent of the sequence of use of individual chemotherapeutic agents, provided that patients are treated with all the active agents (13,14). However, the same principle of maximum exposure and indiscriminate sequence of use of all agents may not be applicable to the use of anti-VEGF and anti-EGFR antibodies (15–17). In the case of cetuximab failure, the option of either switching to a bevacizumab-containing regimen or using cetuximab beyond progression are both practiced but lack supporting evidence. As pre-clinical data have suggested that acquired resistance to anti-EGFR antibody is associated with an increased level of VEGF, the sequence of their use may have practical implications (18–20). In this retrospective study, the outcomes of patients who received bevacizumab-containing regimens following cetuximab failure for Kras wild-type mCRC were reviewed and presented.

## Materials and methods

### Study eligibility

All patients with mCRC who were treated with bevacizumab-containing regimens between January 2006 and December 2011 were screened. Patients were eligible for review in our study if they met the following criteria: i) Kras wild-type mCRC; ii) chemotherapy and cetuximab received as immediate prior treatment; iii) chemotherapy and bevacizumab received as the index line of treatment; and iv) imaging conducted for response evaluation. Out of the 50 patients that were screened, 18 patients satisfied the criteria and were eligible for analysis.

### Chemotherapy regimens

Patients were treated with bevacizumab at 5 mg/kg every 2 weeks if combined with FOLFOX4 (1) or FOLFIRI (irinotecan plus 5-FU and LV) (2), or at 7.5 mg/ kg every 3 weeks if combined with XELOX (capecitabine plus oxaliplatin) (5), XELIRI (capecitabine plus irinotecan) (21) or XELODA (capecitabine alone) (22). No dose adjustment was permitted for bevacizumab, while the dose of chemotherapeutic agents was determined and adjusted at the discretion of the treating oncologist, based on our departmental protocol.

### Outcomes measures

Outcome measures included PFS (from the start of bevacizumab treatment following cetuximab failure, to the first recorded occurrence of physician-assessed disease progression, PD, or death). The objective response rate (ORR) was evaluated by imaging using response evaluation criteria in solid tumors (RECIST) criteria every 8–12 weeks of treatment (23).

Targeted adverse events were recorded in accordance with two prospective observational cohort studies, the BRiTE and BEAT study (24,25). Adverse events included gastrointestinal perforation (GIP; perforation, intra-abdominal abscess and fistula), arterial thromboembolic events (ATEs; myocardial infarction, cerebrovascular accident, transient ischemic attack and unstable angina), postoperative bleeding or wound-healing complications (POWHCs), grade III/IV bleeding and hypertension requiring additional anti-hypertensives. Toxicity grading was based on the National Cancer Institute (NCI) Common Toxicity Criteria for Adverse Events (CTCAE), version 3.0 (26). Adverse events attributed to bevacizumab were identified up to 90 days after permanent discontinuation of the drug.

### Statistical analysis

The primary endpoint of our analysis was median progression-free survival (mPFS) and ORR. Survival rates were estimated using the Kaplan-Meier method and survival curves were compared using the log-rank test. The Fisher’s exact test was used to compare response rates. P<0.05 was considered to indicate a statistically significant difference. Analyses were conducted using the Statistical Package for Social Sciences (SPSS) 19.0 for Windows (SPSS, Inc.; Chicago, IL, USA).

## Results

### Patient characteristics

The median patient age was 56.5 years. Metastatic disease was identified at initial diagnosis in 15 (83.3%) patients, while 10 (55.6%) patients presented with metastases involving more than 1 organ. The liver was the most common site of metastasis and 5 patients exhibited liver-only metastasis (Table I). Following cetuximab failure, 8 and 10 patients received second- and third-line bevacizumab-containing regimens, respectively. Bevacizumab was administered with irinotecan-based chemotherapy in 13 patients and oxaliplatin-based chemotherapy in 5 patients. The median time period from cetuximab failure to the start of bevacizumab treatment was 6.8 weeks (range, 1–60) and the median number of cycles of bevacizumab was 6.5 (range, 4–12).

### Treatment efficacy

**Figure 1.**
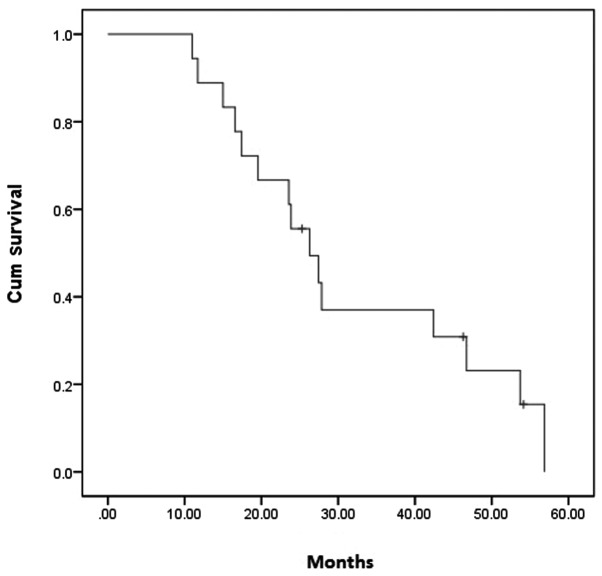
Kaplan-Meier plot of progression-free survival for all patients receiving bevacizumab-containing regimens following cetuximab failure.

**Figure 2.**
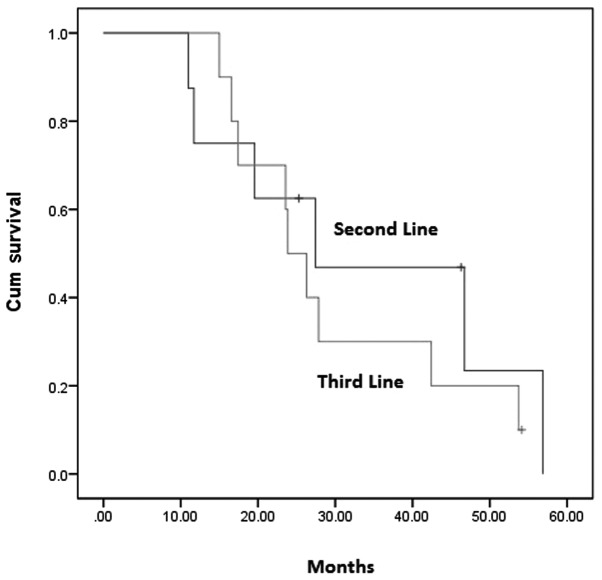
Kaplan-Meier plot of progression-free survival and patients receiving bevacizumab-containing regimens as second- and third-line therapy.

After a median follow-up of 12.1 months, the mPFS for the total group of patients was 26.3 weeks (95% CI, 19.5–33.0) with an ORR of 38.9%. For the 8 patients who received bevacizumab-containing regimens as a second-line treatment, 1 complete response (CR) and 3 partial responses (PR) were observed, producing an ORR of 50%. The mPFS was 27.4 weeks (95% CI, 2.0–52.8). For the 10 patients who received the third-line treatment, 3 PRs were observed and thus the ORR was 30%. The mPFS was 23.9 weeks (95% CI, 19.7–28.1). No statistically significant difference in PFS (P= 0.552) and ORR (P= 0.63) was observed between patients who received bevacizumab as a second- or third-line treatment following cetuximab failure (Figs. 1 and 2).

### Toxicity related to bevacizumab

Two patients presented with worsened hypertension that was controlled by an additional anti-hypertensive drug. One patient was found to have intestinal perforation 78 days after the last dose of bevacizumab. Another patient had bevacizumab suspended for 6 weeks before planned resection of liver and pelvic metastases. Complete resection was achieved and pathological examination confirmed a partial response. Additionally, no post-operative complications were observed for this patient. In the whole cohort of patients, no ATEs or grade III/IV bleeding were observed.

## Discussion

To our knowledge, this is the first study concerning the outcomes of bevacizumab-containing regimens following cetuximab failure in patients with Kras wild-type mCRC.

The treatment outcomes of patients treated with bevacizumab-containing regimens as the second-line therapy were comparable with a previously reported phase III trial (8). Patients treated with bevacizumab-containing regimens as the third-line therapy demonstrated a median PFS of 23.9 weeks and an ORR of 30%, which were both superior to those found previously. Emmanouilides *et al* studied the outcomes of 19 patients who had received bevacizumab with 5-FU plus LV as a third-line treatment in a prospective study. The median time to progression was 16 weeks but no objective response was documented (27). In a retrospective analysis by Kang *et al*, bevacizumab was combined with either FOLFOX or FOLFIRI in a third-line or later treatment after failure of 5-FU, oxaliplatin and irinotecan. The median PFS was 5.3 months and the overall response rate was 9.5% (28). The results of these two studies were similar to those of a TRC-0301 study in which the median PFS was 3.5 months and the response rate was 4% (29). Vincenzi *et al* conducted a phase II study using bevacizumab and 5-FU plus LV as the fourth-line setting in 48 patients who failed cetuximab, oxaliplatin, irinotecan and 5-FU treatment. The response rate was only 6.25%; however, 30.4% of patients achieved a stable disease status (30). In all the aforementioned studies, the Kras status of tumors was not noted and prior cetuximab exposure was only documented in the study by Vincenzi *et al*. Although 17 patients in the present study failed oxaliplatin, 5-FU and cetuximab treatment, only 6 patients in our study as compared with all patients in the aforementioned studies failed oxaliplatin, irinotecan and 5-FU treatment. While ‘chemo-refractoriness’ differed among patients in the present study and those quoted previously, the potential additional benefit of using bevacizumab following cetuximab failure should not be overlooked.

It has been demonstrated that the exact sequence of chemotherapeutic agents used in mCRC chemotherapy did not affect the outcome (14), provided patients were exposed to all active agents (13). However, there is no real evidence for applying the same principle to the use of anti-EGFR and anti-VEGF antibodies. Both the CAIRO2 and PACCE trials demonstrated inferior results with the addition of anti-EGFR antibody to bevacizumab-containing regimens (15,16). These two phase III randomized controlled studies were unable to confirm why administering more did not lead to improvement with regard to the use of targeted therapies; however, the results did call for the investigation of an optimal sequence of use of these targeted therapies. Notably, a study by Norguet *et al* investigated the effect of prior exposure to bevacizumab on the efficacy of subsequent cetuximab treatment (17). In the present study, patients with prior exposure to bevacizumab were associated with a significantly inferior outcome with subsequent cetuximab treatment. Taken together, it is necessary to identify patients who may benefit most from a specific sequence of use of anti-EGFR and anti-VEGF antibodies.

An enhanced treatment effect was demonstrated in patients with Kras wild-type tumor treated with cetuximab (9,12). All patients in the present study had Kras wild-type tumor and were treated with cetuximab. As demonstrated in pre-clinical studies, prolonged exposure of cancer cells to EGFR-blocking antibodies gives rise to resistant cells that have increased VEGF expression. Thus, cancer cells may become more dependent on the VEGF pathway when they acquire resistance to the EGFR inhibitor (18–20). It could be postulated that the superior outcomes of the patients in the present study were partly due to the selection of patients with Kras wild-type tumor; patients were treated with bevacizumab at a time when the cancer cells had become more dependent on the VEGF pathway upon acquiring resistance to the EGFR inhibitor.

In the present study, the toxicity related to bevacizumab was infrequent and manageable. Two patients required administration of one additional anti-hypertensive drug for the treatment of worsened hypertension during the course of bevacizumab. This proportion of patients was similar to those observed in landmark studies with an incidence of 4–11% for grade III/IV hypertension (6–8). The patient who did not survive due to a bowel perforation was unlikely to have suffered the perforation as a result of bevacizumab; the event occurred 78 days after the last dose of bevacizumab when the patient was receiving hypofractionated palliative radiotherapy to the pelvis. Therefore, there were no patient fatalities due to bevacizumab-related toxicity during the active phase of bevacizumab treatment.

In conclusion, the use of bevacizumab-containing regimens following cetuximab failure in patients with Kras wild-type mCRC has modest activity and acceptable toxicity. A small sample size and retrospective nature were the major limitations of the present study. However, the results remain informative. Unlike concurrent use of bevacizumab and cetuximab, and likely the sequential use of cetuximab following bevacizumab, the use of bevacizumab-containing regimens following cetuximab failure may represent an optimal sequence of targeted therapies and warrants further research in prospective studies.

## Figures and Tables

**Table I.** Baseline patient characteristics.

Characteristic	No. patients (n=18)
Median age (range)	56.5 (42–72)
Gender	
Male	9
Female	9
ECOG performance status	
0	3
1	15
Primary tumor site	
Colon	13
Rectum	5
Number of metastatic sites	
1	8
>1	10
Site of metastasis	
Liver	15
Lymph node	8
Lung	5
Locoregional	4
Peritoneum	3
Prior chemotherapy	
Fluoropyrimidine	18
Oxaliplatin	17
Irinotecan	6
